# TLR2 and TLR4 Are Expressed in Epiretinal Membranes: Possible Links with Vitreous Levels of Complement Fragments and DAMP-Related Proteins

**DOI:** 10.3390/ijms25147732

**Published:** 2024-07-15

**Authors:** Lucia Dinice, Graziana Esposito, Andrea Cacciamani, Bijorn Omar Balzamino, Pamela Cosimi, Concetta Cafiero, Guido Ripandelli, Alessandra Micera

**Affiliations:** 1 Research and Development Laboratory for Biochemical, Molecular and Cellular Applications in Ophthalmological Science, IRCCS—Fondazione Bietti, 00184 Rome, Italy; lucia.dinice@fondazionebietti.it (L.D.); graziana.esposito@fondazionebietti.it (G.E.); bijorn.balzamino@fondazionebietti.it (B.O.B.); 2 Surgical Retina Research Unit, IRCCS—Fondazione Bietti, 00184 Rome, Italy; andrea_cacciamani@hotmail.com (A.C.); pcosimi@gmail.com (P.C.); guido.ripandelli@fondazionebietti.it (G.R.); 3 Anatomic Pathology Unit, Fabrizio Spaziani Hospital, 03100 Frosinone, Italy; concettacafiero@gmail.com

**Keywords:** toll-like receptors (TLRs), epiretinal membranes (ERMs), vitreous, complement fragments, extra-cellular matrix (ECM), innate immunity, inflammation, tissue remodeling, vitreoretinal diseases

## Abstract

Previous studies reported the expression of toll-like receptors (TLRs), merely TLR2 and TLR4, and complement fragments (C3a, C5b9) in vitreoretinal disorders. Other than pathogens, TLRs can recognize endogenous products of tissue remodeling as damage-associated molecular pattern (DAMPs). The aim of this study was to confirm the expression of TLR2 and TLR4 in the fibrocellular membranes and vitreal fluids (soluble TLRs) of patients suffering of epiretinal membranes (ERMs) and assess their association with disease severity, complement fragments and inflammatory profiles. Twenty (n = 20) ERMs and twelve (n = 12) vitreous samples were collected at the time of the vitrectomy. Different severity-staged ERMs were processed for: immunolocalization (IF), transcriptomic (RT-PCR) and proteomics (ELISA, IP/WB, Protein Chip Array) analysis. The investigation of targets included TLR2, TLR4, C3a, C5b9, a few selected inflammatory biomarkers (Eotaxin-2, Rantes, Vascular Endothelial Growth Factor (VEGFA), Vascular Endothelial Growth Factor receptor (VEGFR2), Interferon-γ (IFNγ), Interleukin (IL1β, IL12p40/p70)) and a restricted panel of matrix enzymes (Matrix metalloproteinases (MMPs)/Tissue Inhibitor of Metallo-Proteinases (TIMPs)). A reduced cellularity was observed as function of ERM severity. TLR2, TLR4 and myD88 transcripts/proteins were detected in membranes and decreased upon disease severity. The levels of soluble TLR2 and TLR4, as well as C3a, C5b9, Eotaxin-2, Rantes, VEGFA, VEGFR2, IFNγ, IL1β, IL12p40/p70, MMP7 and TIMP2 levels were changed in vitreal samples. Significant correlations were observed between TLRs and complement fragments and between TLRs and some inflammatory mediators. Our findings pointed at TLR2 and TLR4 over-expression at early stages of ERM formation, suggesting the participation of the local immune response in the severity of disease. These activations at the early-stage of ERM formation suggest a potential persistence of innate immune response in the early phases of fibrocellular membrane formation.

## 1. Introduction

The epiretinal membranes (ERMs), also known as macular pucker or cellophane maculopathy, frequently develop with ageing and can be associated with diabetes, cardiovascular disorders and metabolic syndrome [1]. Structurally, these avascular fibrocellular membranes are layered over the retina, in the macular zone, and contract the vitreoretinal interface, depending on the profibrogenic activity [1].

Several extra-retinal cells (T/B lymphocytes (CD4^+^, CD8^+^, CD22^+^), macrophages (CD68^+^), neutrophils and several MHC class II cell surface receptor (HLA-DR)-expressing cells [2,3] and a few retinal cell types (Müller cells, astrocytes, hyalocytes, retinal pigment epithelium cells and fibroblasts/myofibroblasts) were observed in whole flattened ERMs [4]. The contraction properties, depending on cell recruitment and extracellular matrix (ECM) synthesis/deposition (matrix remodeling), are regulated by local cell-to-ECM interactions and a plethora of proteins (growth factors, neuromediators and matrix-linked enzymes) [5]. This microenvironment triggers a tractional force between the membrane and retina (vitreoretinal interface), as enriched of the main profibrogenic factors, such as transforming growth factor β1 (TGFβ1), glial cell-derived growth factor (GDNF), vascular endothelial growth factor (VEGF) and neurotrophins (nerve growth factor (NGF), brain-derived neurotrophic factor (BDNF)) [4]. This microenvironment is the result of different cell types (activated Müller cells, contractile myofibroblasts (myoFBs) and reactive microglia [6,7]) and myofibroblast-like cells that participate in the synthesis of ECM and the release of growth factors/cytokines, influencing the contractile abilities of the membrane and contributing to disease pathogenesis (inflammation and angiogenesis) [4,8]. Müller cells, hyalocytes and retinal pigment epithelium-derived cells have been identified as potential myofibroblast precursors [4]. We previously analyzed the protein profile of vitreous and vitreal reflux collected, respectively, at the time of the vitrectomy and intravitreal anti-VEGF injection, showing that information about vitreal protein profiles might mirror of health of underneath retina, retinal ganglion cell (RGC) and/or photoreceptors [7,8,9].

The long-lasting ECM makeover (tissue remodeling) seems frequently associated with the production, release and accumulation of molecules associated with endogenous cellular and tissue stress signals (the Damage-Associated Molecular Patterns (DAMPs) and alarmins) [10]. Tissue-injury molecules (collagen and hyaluronan fragments) can be generated inside the fibrocellular membranes upon matrix retraction [11]. Accumulating inside tissues or being released in biological fluids, DAMPs might trigger a potent inflammatory response during non-infection-driven inflammation [10]. ECM fragments are sensed by toll-like receptors (TLRs) expressed by epithelial cells, innate-immune cells, antigen-presenting cells [12]. By allowing a specific recognition of Pathogen Associated Molecular Patterns, (PAMPs) and endogenous molecules (DAMPs), such as death-cell debris, intact or fragmented collagen and hyaluronic acid, inflammatory products and oxidative-modified lipids, TLRs prompt a local innate immune response and guarantee immune surveillance, allowing tissue homeostasis [12,13,14,15]. DAMPs preferentially interact with TLR2, TLR4 and complement cleaved fragments (C3a, C5b), in both membrane-bound or soluble forms [16,17,18,19,20,21,22]. Soluble TLRs and complement fragments accumulate in blood, urine, saliva, tears and aqueous and vitreous humors, as observed in infections and chronic autoimmune disorders [23]. Signals from innate immune response activation have been described in diabetic retinopathy (DR), age-related macular degeneration (AMD) and macular pucker (ERMs) [2,24]. The concept of functional cross-talk between TLRs and complement fragments has been accurately discussed in a recent revision of literature [23].

Therefore, the aim of the present study was to verify the expression of TLR2 and TLR4 in all the membranogenic phases of ERM disease, by means of immunolocalization (ERMs) and biomolecular analysis (ERMs/vitreous). The association between a few selected complement fragments (C3a, C5b-9) and inflammatory/remodeling mediators potentially expressed by these fibrocellular membranes was also investigated.

## 2. Results

In this observational single-point study, all ERMs (n = 20) and paired vitreal fluids were categorized as stage 2, stage 3 and stage 4 of the disease. The categorization was carried out by the surgeon at the time of clinical assessment before vitreoretinal surgery (Optical coherence tomography (OCT) bioinstrumental evaluation; Govetto’s nomenclature) [25,26].

The characteristics of the study population are shown in Table 1. Due to the features of the diseases and the absence of controls, stage 1 was not included in this analysis, and stage 2 was used as the referring control for comparative studies, depending on the analysis.

This small study population displayed different comorbidities, in line with age and disease features, distributed as follows: 50% (10/20) comorbidities with hypertension and 35% (7/20) comorbidities without hypertension. A total of 15% (3/20) declared no comorbidities of interest for this study (none, apparent good health). 

### 2.1. TLR2 and TLR4 Immunoreactivity in ERMs

**Figure 1:**
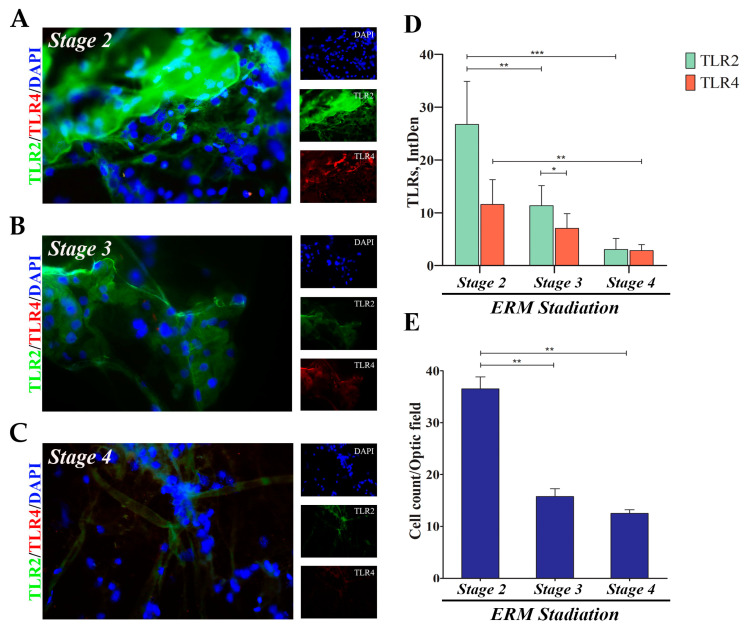
TLR2 and TLR4 protein expression in ERMs as a function of disease severity. A total of six membranes (N = 2 for stage 2, 1F/1M; N = 2 for stage 3, 1F/1M; N = 2 for stage 4, 1F/1M) were collected during pars plana vitrectomy and processed for Epifluorescent analysis (**A**–**C**), coupled to Integrated Density (IntDen; (**D**)) evaluation. Representative merged (main) and single (right side) panels of TLR2 (green) and TLR4 (red) in ERMs at stage 2 (**A**), stage 3 (**B**) and stage 4 (**C**). Membranes were counterstained with nuclear DAPI (blue) to better visualize the cells. Note the significant decrease in immunoreactivity (**D**) and cellularity (**E**), depending on severity (*p* ≤ 0.05). Data represent mean ± SEM and values of fluorescent intensity (IntDen; ImageJ) are expressed in arbitrary units. Magnifications ×400 (bar size = 50 µm). Significances are shown in the panels (* *p* ≤ 0.05; ** *p* ≤ 0.01; *** *p* ≤ 0.005), as calculated using one-way ANOVA followed by a Tukey–Kramer post hoc test (mean ± SEM).

A widespread nuclear staining (DAPI/blue) was depicted in all ERMs, indicating the presence of several cell subsets embedded in the fibrous matrix (fibrocellular scaffolds). As shown in Figure 1, specific TLR2 (green/cy2) and TLR4 (red/cy3) immunoreactive cells were observed in ERMs at all stages (×400). Particularly, high TLR2 and moderate TLR4 expression characterize stage 2 (Figure 1A) with respect to stage 3 (Figure 1B) and stage 4 (Figure 1C). This decreasing trend was confirmed by imaging quantification. As shown in Figure 1D, the fluorescent analysis (IntDen) specific for each target showed higher TLR2 and TLR4 expression at early stages of the disease, with TLR2 IntDen being higher than TLR4. Of interest, ERM cellularity decreased with increasing severity, along with matrix deposition. Figure 1E shows the reduction in cellularity with increasing ERM severity, implying that the ERMs at stage 4 had less cellularity than at stage 3 and stage 2.

### 2.2. TLR2 and TLR4 Immunoreactivity: Alpha-Smooth Muscle Actin (α-SMA)-Fibroblast like Cells, Glial Fibrillary Acidic Protein (GFAP)-Activated Müller Cells and Ionized Calcium-Binding Adapter Molecule 1 (Iba1)-Bearing Ameboid Microglia

**Figure 2:**
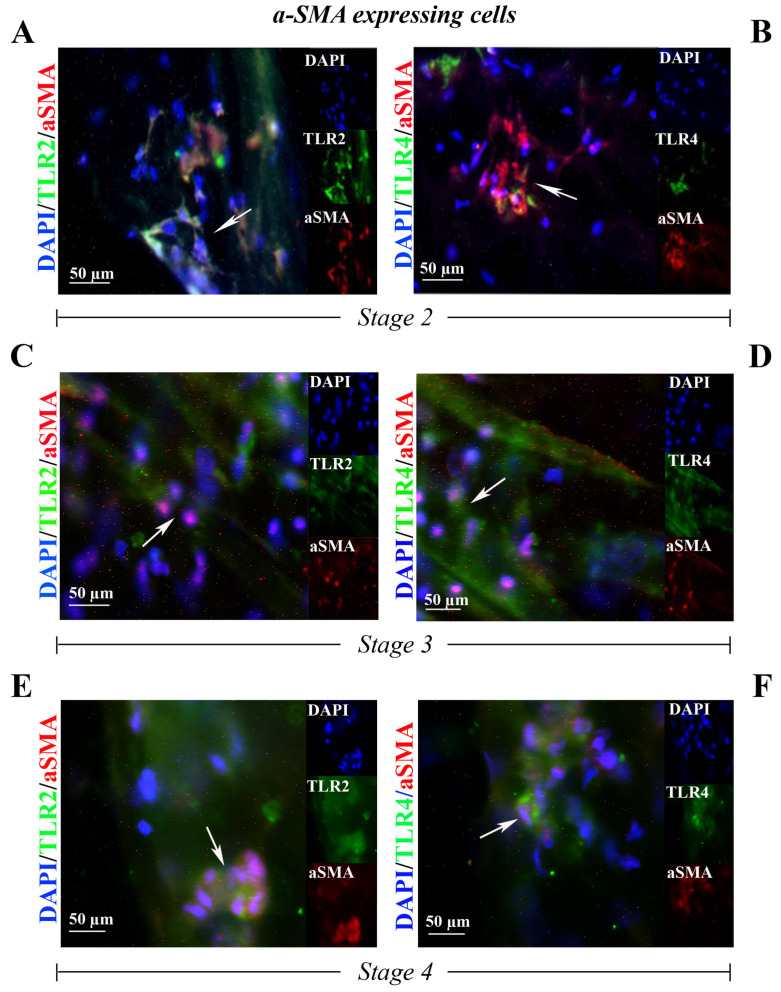
TLR2 and TLR4 immunoreactivity in myoFBs cells populating the ERM at stage 2 ((**A**,**B**) N = 2, 1F/1M), at stage 3 ((**C**,**D**) N = 2, 2F) and at stage 4 ((**E**,**F**) N = 2, 2F). Representative fluorescent panels of TLR2/green (**A**,**C**,**E**) or TLR4/green (**B**,**D**,**F**) and α-SMA/red from double-immunostained and nuclear counterstained (DAPI/blue) ERMs. Note the TLR2 and TLR4 immunoreactivity in, respectively, α-SMA positive myoFBs. Citofixed and whole-mounted membranes were used. Merged and single-channel panels are shown. Magnifications ×400 (bar size = 50 µm). Arrows indicate coexpression of targets.

The nuclear staining (DAPI/blue) of peeled-off ERM tissues highlighted the presence of several cell subsets inside the fibrocellular-matrix compartment of ERMs, as reported in previous studies. A specific TLR2 (Figure 2A,C,E) and TLR4 (Figure 2B,D,F) expression was observed in α-SMA positive myoFBs-like cells populating the whole flattened ERMs at stage 2 (Figure 2A,B), at stage 3 (Figure 2C,D) and at stage 4 (Figure 2E,F). 

**Figure 3:**
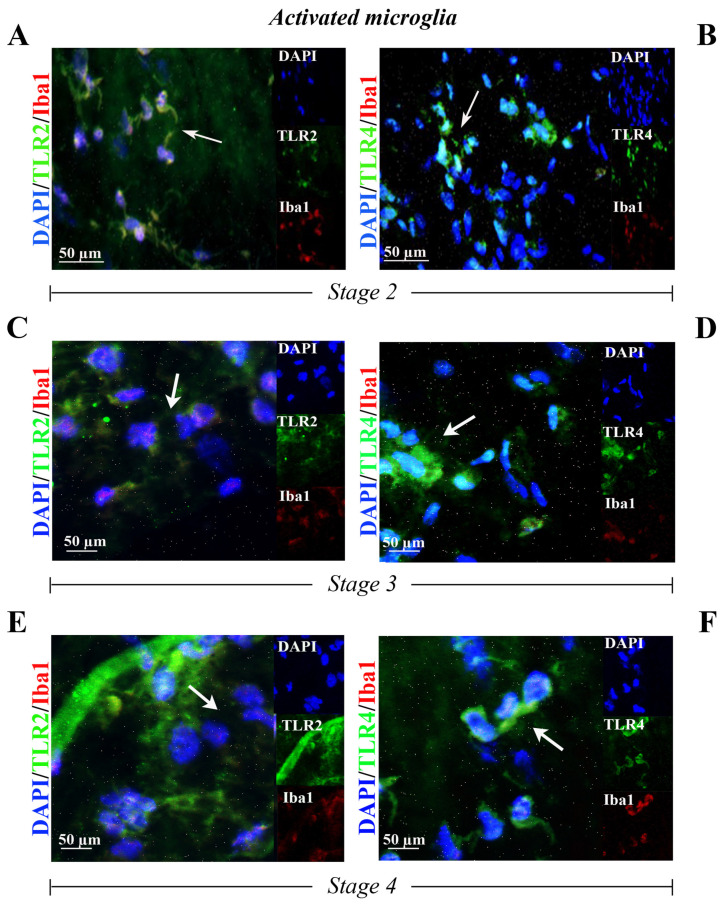
TLR2 and TLR4 immunoreactivity in reactive microglia cells populating the ERM at stage 2 ((**A**,**B**) N = 2, 2F), at stage 3 ((**C**,**D**) N = 1, 1F) and at stage 4 ((**E**,**F**) N = 1, 1F). Representative fluorescent panels of TLR2/green (**A**,**C**,**E**) or TLR4/green (**B**,**D**,**F**) and Iba1/red from double-immunostained and nuclear counterstained (DAPI/blue) ERMs. Note the TLR2 and TLR4 immunoreactivity in, respectively, some Iba1 immunoreactive cells. Citofixed and whole-mounted membranes were used. Merged and single-channel panels are shown. Magnifications ×400 (bar size = 50 µm). Arrows indicate coexpression of targets.

Immunoreactivity for TLR2 and TLR4 was observed in the Iba1-positive cells (respectively, Figure 3A,C,E and Figure 3B,D,F) in ERMs at stage 2 (Figure 3A,B), at stage 3 (Figure 3C,D) and at stage 4 (Figure 3E,F). 

**Figure 4:**
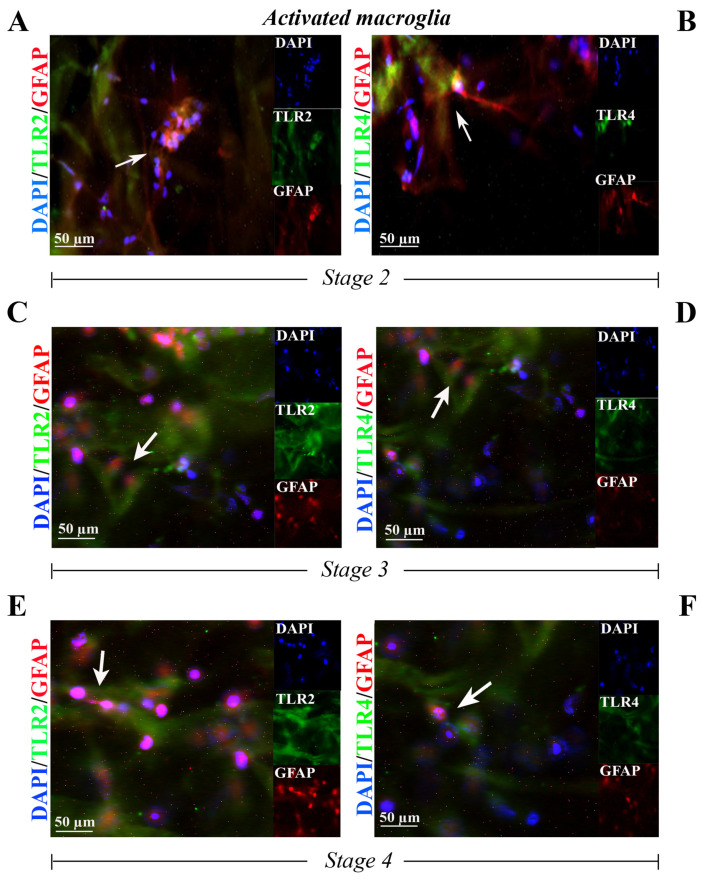
TLR2 and TLR4 immunoreactivity in activated Müller cells populating the ERMs at stage 2 ((**A**,**B**) N = 2, 2F), at stage 3 ((**C**,**D**) N = 1, 1F) and at stage 4 ((**E**,**F**) N = 1, 1F). Representative fluorescent panels of TLR2/green (**A**,**C**,**E**) or TLR4/green (**B**,**D**,**F**) and GFAP/red and nuclear counterstained (DAPI/blue) ERMs. Note the TLR2 and TLR4 immunoreactivity in, respectively, some cellular compartments of GFAP-positive Müller cells. Citofixed and whole-mounted membranes were used. Merged and single-channel panels are shown. Magnifications ×400 (bar size = 50 µm). Arrows indicate coexpression of targets.

Finally, a specific TLR2 (Figure 4A,C,E) and TLR4 (Figure 4B,D,F) expression was observed in GFAP-positive cells inside the whole flattened ERMs at stage 2 (Figure 4A,B), at stage 3 (Figure 4C,D) and at stage 4 (Figure 4E,F).

### 2.3. TLR2/TLR4/myD88 Transcripts Are Increased in ERMs as a Function of Disease Severity

**Figure 5:**
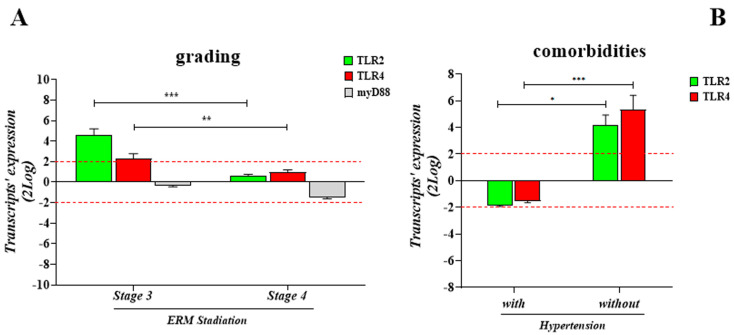
Transcripts’ expression as a function of disease severity and comorbidities. (**A**) Histogram showing the significant upregulation of *TLR2*mRNA in ERM at stage 3 with respect to stage 4, as well as compared to stage 2. The same trend was observed for *TLR4*mRNA. An inverted trend was observed for the adaptor molecule *myD88*mRNA, as downregulation was observed in ERM at stage 4 with respect to stage 3, and no significant changes were observed with respect to stage 2. Values are relative expression ratios (fold-changes in log2-scale; mean ± SEM), as generated by REST-analysis, comparing stage 3 or stage 4 with respect to stage 2. (**B**) Transcriptomic analysis highlighted a decreased *TLR2* and *TLR4* expression in the presence of the major comorbidities associated with hypertension (10/20; ID: 1, 2, 4, 8, 12, 13, 14, 15, 17, 19), while an increased expression was detected in the absence of hypertension (7/20; ID: 3, 5, 9, 10, 11, 16, 18). Values are relative expression ratios (fold-changes in log2-scale; mean ± SEM), as generated by REST-analysis, comparing clusters vs. normal controls (3/20; IDs: 3, 5, 10). The *p*-values were calculated according to the REST-ANOVA Tukey–Kramer coupled analysis. Red dot lines are referred to 2log-FC PCR biological significance. Significances are shown in the panels (* *p* ≤ 0.05; ** *p* ≤ 0.01; *** *p* ≤ 0.005), as calculated using one-way ANOVA followed by a Tukey–Kramer post hoc test (mean ± SEM).

The transcription activity of *TLR2* and *TLR4* was verified by relative RT-PCR, considering the expression in stage 3 and stage 4 ERMs with respect to stage 2 (referred to as 1). As shown in Figure 5A, the relative analysis showed a significative upregulation of the *TLR2* transcript at stage 3 (4.59 ± 1.72 2logFC) and a trend toward an increase at stage 4 (0.65 ± 0.31 2logFC), with respect to stage 2. A slight upregulation of *TLR4* was detected at stage 3 (2.28 ± 1.41 2logFC) and a slight trend toward an increase was observed at stage 4 (0.96 ± 0.64 2logFC), as compared to stage 2. Regarding the specific adaptor molecule, a trend toward a decrease in the myD88 transcript expression was observed at stage 3 (−0.33 ± 0.36 2log-ratio; *p* > 0.05) and at stage 4 (−1.49 ± 0.39 2log-ratio; *p* > 0.05), with respect to stage 2 (referred to as 1) (Figure 5A).

For assessing the possibility that comorbidities might influence this transcription profile, a further clustering was carried out, considering comorbidities with and without hypertension, as shown in Figure 5B. Notably, *TLR2* and *TLR4* relative transcript expression was significantly higher in the ERMs from comorbidities without hypertension (+4.198 ± 0.735 FC 2log-ratio for *TLR2*; +5.331 ± 1.090 FC 2log-ratio for *TLR4*) and lower in comorbidities with hypertension (−1.869 ± 0.0735 FC 2log-ratio for *TLR2*; −1.528 ± 0.123 FC 2log-ratio for *TLR4*). Data were found with respect to same-stage ERMs obtained from patients without comorbidities (none).

### 2.4. The Soluble Forms of TLR2 and TLR4 Are Detected in Vitreous Samples

**Figure 6:**
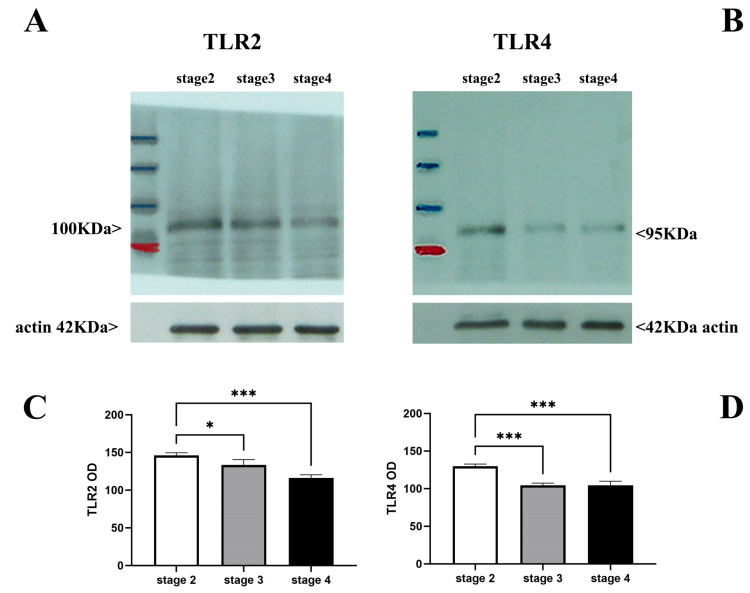
TLR2 and TLR4 levels in vitreous as a function of disease severity. A total of twelve specimens: N = 5 for stage 2 (ID: 2, 11, 13, 14, 19); N = 4 for stage 3 (ID: 8, 15, 16, 17); N = 3 for stage 4 (ID: 1, 4, 10) were processed for biochemical analysis. Briefly, the untouched vitreous samples were sonicated and cleared for Western blotting analysis. Representative vitreal levels of TLR2 and TLR4 (respectively, (**A**,**B**); IP/WB) are shown by gels, and densitometric IntDen analysis (**C**,**D**) was carried out considering all the samples. Note that TLR2 and TLR4 levels are comparable at early stages and greater than levels at late stages of the disease. Protein normalization was confirmed by actin expression. Data are mean ± SEM from optical density (OD) analysis (ImageJ) and *p*-values are shown by asterisks (see Section 4). Significant levels are shown in the panels (* *p* ≤ 0.05; *** *p* ≤ 0.005), as calculated using one-way ANOVA followed by a Tukey–Kramer post hoc test (mean ± SEM).

Immunoprecipitation, coupled to Western blot analysis (IP/WB), was used for quantifying the presence of soluble TLR2 and TLR4 proteins. The quantification of TLR2 (Figure 6A,C) and TLR4 (Figure 6B,D) in vitreous samples showed that both TLRs are less expressed at late stages and more highly expressed in the early stages of disease. 

### 2.5. TLR2 and TLR4 Correlates with Biomarkers of Inflammation and Tissue Matrix Remodeling

An interesting aspect of the TLRs activation is the downstream signaling leading to the release of pro-inflammatory/angiogenic mediators (HLA-DR, p65-NFkB, Eotaxin-2, Rantes and VEGFA/VEGFR2) and matrix remodeling enzymes (MMPs/TIMPs), modulating antibacterial, antimicrobial and immune adaptive and apoptotic responses [27,28,29,30]. 

**Figure 7:**
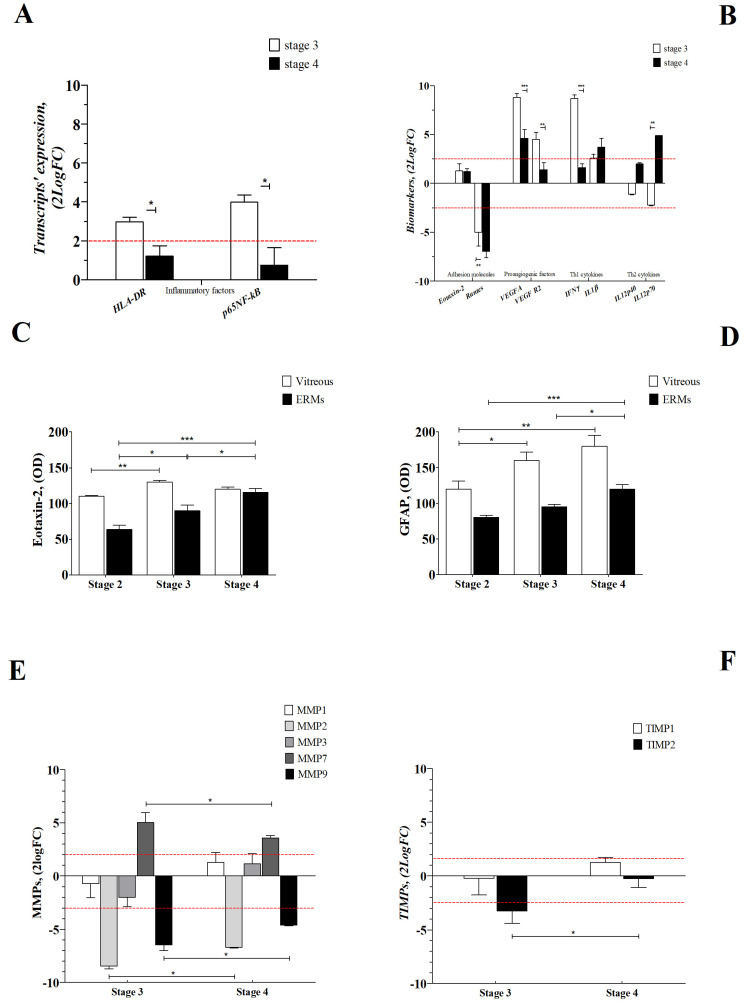
Inflammatory and remodeling profiles. (**A**,**B**) Inflammatory (**A**) and adhesion, proangiogenic and Th1/Th2 (**B**) patterns were analyzed, respectively, in ERMs and vitreous samples. (**A**) Note the *HLA-DR* and *p65NFkB* transcripts’ upregulation in ERMs at stage 3. (**B**) Adhesion molecules appear deregulated, *VEGFA* and *VEGFR2* were upregulated at stage 3, and only *IL1β* and *IL12p70* were increased at stage 4, all in vitreous samples. (**C**,**D**) Eotaxin-2 (**C**) and GFAP (**D**) expression in vitreal samples and coupled ERMs, as assayed by IP/WB analysis. Note that Eotaxin-2 and GFAP were increased in both membrane formation and coupled vitreous, in a manner related to disease severity. All diseases stages are shown. (**E**,**F**) Histogram depicting the relative transcript expression of *MMPs* (**E**) and *TIMPs* (**F**) in ERM extracts as function disease severity. Note the *MMP2* and *MMP9* transcript downregulation and the *MMP7* transcript upregulation as early as stage 3. Whenever required, fold changes were calculated with respect to stage 2, and comparisons were carried out between stages. Data in the bar graph are shown as mean ± SEM (2log fold-changes) and *p*-values are shown by asterisks (see Section 4). Red dot lines are referred to 2log-FC PCR biological significance and significant levels are shown in the panels (* *p* ≤ 0.05; ** *p* ≤ 0.01; *** *p* ≤ 0.005), as calculated using one-way ANOVA followed by a Tukey–Kramer post hoc test (mean ± SEM).

Figure 7A highlights the upregulations of *HLA-DR* and nuclear factor kappa-light-chain-enhancer of activated B cells (*p65-NFkB*) transcripts at stage 3 in ERMs, and a positive correlation between HLA-DR and respectively *TLR2* (rho = 0.978, *p* < 0.04) and *TLR4* (rho = 0.979, *p* < 0.005) was also observed. In these ERMs, as expected, the increased *p65-NFkB* transcripts expression observed at stage 3 were decreased at stage 4 (Figure 7A). Both *Eotaxin-2* and *Rantes* were decreased, while *VEGFA* and *VEGFR2* increased at stage 3 but were not retained at stage 4 in vitreous samples (Figure 7B). Regarding the vitreal Th1/Th2 profile, while *IFNγ* was reduced with ERM severity, *IL1β* and *IL12p70* increased depending on severity (Figure 7B). 

The increased Eotaxin-2 levels detected in vitreal fluids, as confirmed by immunoprecipitation and immunoblotting (IP/WB) analysis, were not consistent with those detected in ERMs (Figure 7C). GFAP protein levels in both vitreous and membranes were increased, depending on ERM stadiation (Figure 7D), confirming the presence of active gliosis both locally and circulating the vitreal chamber. 

A transcriptomic analysis specific for *MMP1, MMP2, MMP3, MMP7, MMP9, TIMP1* and *TIMP2* in ERM extracts showed that only *MMP7* was significantly changed at stage 3 and stage 4 (Figure 7E). On the contrary, the *MMP2* and *MMP9* transcripts were significantly deregulated at stage 3, while the *MMP1* and *MMP3* transcripts were unchanged (Figure 7E). With respect to their tissue regulators, only *TIMP2* transcripts were decreased at stage 3 (Figure 7F). A strong positive correlation was detected between *MMP7* and *TLR2* at stage 3 (rho = 0.9948, *p* < 0.05). 

### 2.6. TLR2 and TLR4 Correlate with Complement Fragments

**Figure 8:**
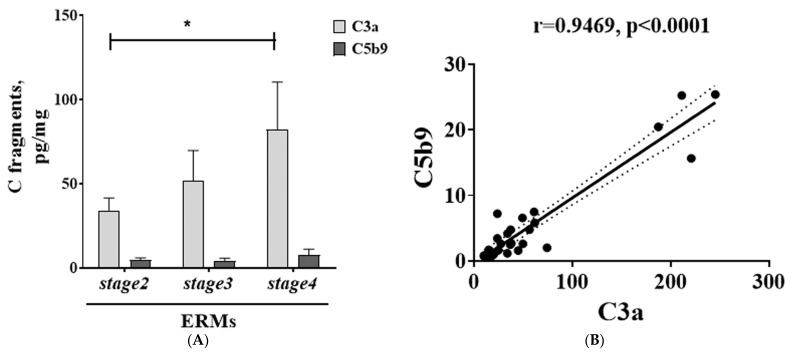
Complement fragments in vitreal fluids as function of ERM severity. Vitreal levels of C3a and C5b9 (ELISA; (**A**)); note that C3a levels were higher than C5b9 levels. (**B**) Scatterplot displaying the positive relationship between C3a and C5b9 markers. Pearson correlation coefficient (r) and significance are shown in the panel. Data are mean ± SEM, as detected by ELISA assay. Significant levels are shown in the panels (* *p* ≤ 0.05), as calculated using one-way ANOVA followed by a Tukey–Kramer post hoc test (mean ± SEM).

Both the TLR family and complement system are main branches of innate immunity [31]. As shown in Figure 8A, increased levels of the soluble forms of C3a were quantified in the vitreous, as function of ERM severity. Higher C3a levels were observed at stage 4. By contrast, no significant changes were monitored for the vitreal C5b9 fragment. Unexpectedly, a positive correlation was observed between C3a and C5b9 markers (Figure 8B).

**Figure 9:**
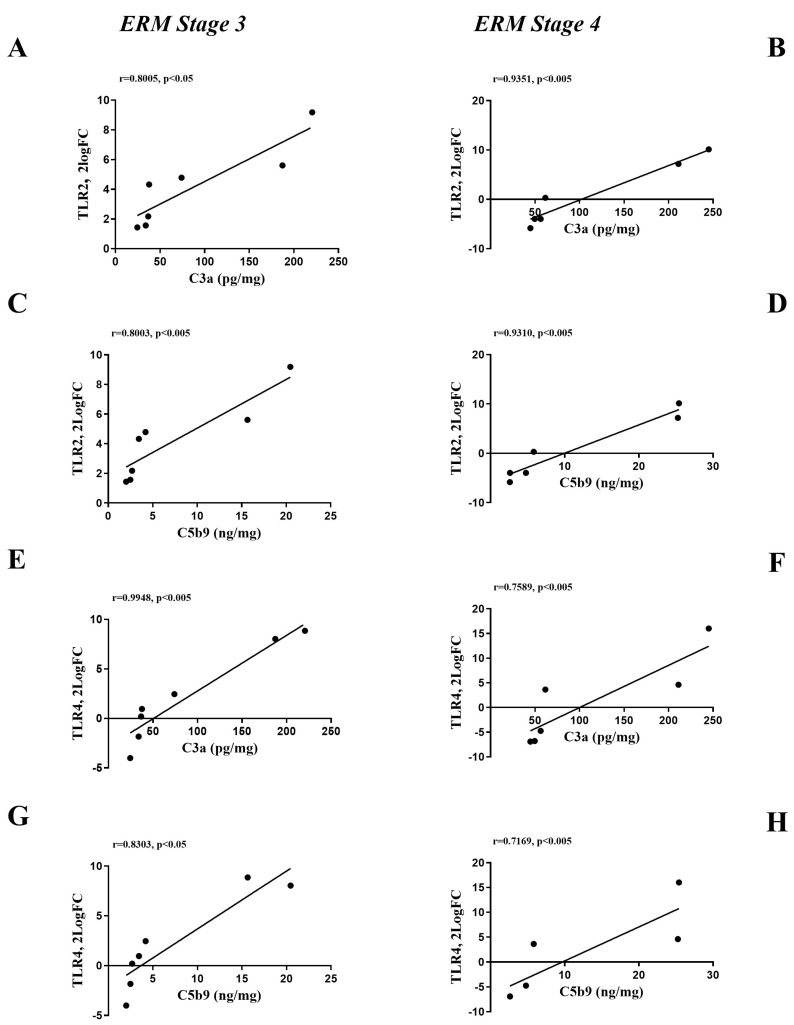
Correlation analysis for *TLRs* (transcripts) and complement fragments (vitreal levels) depending on ERM severity. Scatterplot specific for *TLR2*/C3a at stage 3 (**A**) and stage 4 (**B**); *TLR2*/C5b9 at stage 3 (**C**) and stage 4 (**D**); for *TLR4*/C3a at stage 3 (**E**) and stage 4 (**F**); for *TLR4*/C5b9 at stage 3 (**G**) and stage 4 (**H**). Note the positive correlation between complement fragments and *TLR2* and *TLR4*. The result of Pearson correlation analysis is shown inside each panel (correlation coefficient, r, and significance).

By correlating the *TLRs* transcripts and vitreal complement values, positive correlations were detected between *TLR2*-C3a (Figure 9A,B), *TLR2*-C5b9 (Figure 9C,D), *TLR4*-C3a (Figure 9E,F) and *TLR4*-C5b9 (Figure 9G,H) at stage 3 and stage 4.

## 3. Discussion

Herein, we show the specific immunoreactivity of TLR2 and TLR4 in ERMs and their immunolocalization to GFAP-, Iba1- and α-SMA-expressing cells. TLR2 and TLR4 also correlated with C3b and C5b9 and some inflammatory mediators. The rationale behind TLR2 and TLR4 expression is discussed below. 

Previous studies emphasized the involvement of membrane-associated and soluble TLRs in retinal degeneration diseases, as shown by in vitro/in vivo and human ex vivo studies (iatrogenic, diabetic, vascular and age-related vitreoretinal diseases) [32,33,34,35,36,37]. Particularly, TLR2 and TLR4 were associated with innate immune activation of complement fragments, promoting the phagocytic activities of mononuclear cells and oxidative stress-mediated responses [31,38,39]. This study extended the previous knowledge on TLR2 and TLR4 on ERMs by hypothesizing their expression in these fibrocellular membranes (ERMs) and coupled vitreal fluids, exploring the TLR2-TLR4’s contribution to ERMs’ severity. This hypothesis is sustained by the following: i. the presence of some cells of immune-derivation (Müller, astrocytes and microglia) inside the membrane and ii. the ability of TLRs to recognize endogenous danger molecules (DAMPs) released from the extracellular and intracellular space of damaged tissue or dead cells (collagen and hyaluronic acid fragments, hyaluronan, heat shock protein (HSP60), fibronectin and other tissue products) [32]. Activated DAMPs might trigger an uncontrolled and unbalanced innate immune response, contributing to and/or exacerbating the ERM-mediated retraction of the underneath retina [40].

First, the TLR2 and TLR4 immunofluorescence was inversely proportional to disease severity (ERM grading), and specific immunoreactivity for TLR2 and TLR4 was restricted to GFAP-reactive Müller cells, Iba1-bearing ameboid microglial cells and α-SMA-expressing cells (myofibroblast-like cells). These cells are known to recognize DAMPs exacerbating the local inflammation in the presence of matrix fragments/debris [41,42,43,44,45,46,47]. Previous studies also referred to activated Müller cells and reactive microglia as major cell sensor patterns in ERMs, and the myofibroblast-like cells’ contribution was related to long-lasting ERM retraction [48]. The differential expression of TLR2 and TLR4 alongside the ERM phases of severity might be linked to the fact that Müller cells seem to play a primary role in the events leading to ERM formation (known as vertical gliosis) [48]. The impact of vertical traction on Müller cells might drive matrix synthesis and scaffold creation, paving the way for migration and cell organization of ERMs. In addition, these fibrocellular membranes confer resistance to the mechanical stimulation of the retina [48]. Our observation of TLR4 immunoreactive microglia is in line with previous studies, and no other TLRs, except for TLR2 and TLR4, have been detected on retinal microglia [49,50,51]. On the contrary, the observation of TLR2 and TLR4 immunoreactivity in activated GFAP-expressing Müller cells is in line with Kumar and Shamsuddin’s findings, reporting the presence of TLR2 and TLR4 in retinal Müller cells [46,52]. Since some in vitro simulations highlighted the ability of TLR2 and TLR4 agonists to trigger the release of IL-6 and MIP-2/C-X-C Motif Chemokine Ligand (CXCL2) chemokines by reactive Müller cells, our observations in ERMs might suggest that TLRs expressing Müller cells can drive immunoregulatory functions in vitreoretinal diseases through DAMP products, in addition to the known role in anti-microbial surveillance (endophthalmitis) [46]. More interestingly, the presence of TLR-bearing myofibroblast-like cells might suggest a possible influence of DAMP-related TLRs on the contractile activities of these fibrocellular-matrix formations [45,46,47]. Collagen fragments might exacerbate the force generated by the fibrocellular membranes on the underneath retina. Precisely, type-II collagen can allow the jelly-like vitreous to adhere to the underneath retina, although ageing or traumatic/inflammatory events can trigger a gradual change of this jelly fluid, inducing its separation from retina (vitreous detachment) [53].

Our previous studies highlighted the existence of specific “ERM-vitreal protein-print” depending on the severity and origin of vitreoretinal damage, highlighting the possibility to use this protein signature for individualized therapy purposes [6]. A significant increase in TLR2 and TLR4 expression suggests the following: i. the activation of intracellular signaling, with p65NFkB nuclear translocation; ii. a huge cell-mediated response driven by professional cells (Antigen-Presenting Cell (APCs), phagocytes), accelerating the local inflammation by the adhesion of leukocytes and the maturation/migration of immune cells; and iii. RGC damage, with an exacerbation of local inflammation and impairments in the retinal network [54]. Herein, the observation of myD88 deregulation clearly indicates the presence of a less-activated pathway related to pro-inflammatory mediators (IFNγ, IL6, IL8), as reported by several in vivo and in vitro myD88 models [55].

Since a decreased cellularity occurs in late stages of the disease (ERM severity), another aspect of TLR2 and TLR4 signaling might be to drive the death of subsequent necrotic cells and APCs by means of NFκB signaling in a paracrine/autocrine fashion, as reported by in vitro simulations [56]. Under physiological processes, the removal of debris and dead cells is essential for protecting retinal networking, as documented by several experimental and ex vivo studies [57]. The apoptosis of myofibroblast-like cells might also occur in the later stages of ERM activity, supporting the reduced retraction intensity observed at stage 4, which often does not require ERM peel-off [25]. Our data on TLRs and HLA-DR expression are supported by previous studies reporting the specific cooperation of HLA-DR with TLR2 and TLR4 [58]. 

An interesting aspect of the TLR activation is the downstream signaling, leading to the release of pro-inflammatory/angiogenic and matrix enzymes able to modulate antibacterial, antimicrobial, as well as adaptive response and apoptotic response [27,28,29,30].

Our investigations in vitreal-ERM paired samples also highlighted an increase in Eotaxin-2 proteins in ERMs and vitreal fluids, in line with previous studies, anticipating the higher Eotaxin-2 levels in vitreous collected from retinal detachment and ERMs [59,60,61]. Of interest, the quantification of GFAP protein in these samples might corroborate the findings on activated Müller cells and their possible loss in the severe forms of the disease [62]. 

A balanced regulation between MMPs and TIMPs is essential in controlling the innate host defense and tissue repair/homeostasis [63]. Under a chronic inflammatory phenotype, TLRs can drive the matrix makeover via the modulation of specific enzymes (MMPs and TIMPs) [64]. Our transcriptomic analysis for MMPs/TIMPs enzymes showed that MMP7 and TIMP2 were significantly modulated in ERMs. A possible connection between MMP and TLRs could be envisaged, given that a strong positive correlation was detected between MMP7 and TLR2.

The Pattern Recognition Receptors (PRRs), including toll-like receptors (TLRs) and complement receptors (CRs), regulate the processes of recognition of pathogens/PAMPs and their clearance [65]. A strong interaction between complement fragments and TLR signaling has been reported in previous in vivo studies, suggesting a novel mechanism by which the complement system might promote inflammation and modulate adaptive immunity in tissues [31]. Activated complement fragments are crucial mediators of inflammation, and the complement cascade activation can actively mediate the inflammatory process working on leucocytes’ recruitment and activation [66,67,68]. The presence of C5b9 complexes, some complement regulators, and abundant cytokines has been discussed in retinal diseases [69]. The innate response is highly influenced by some complement components (C3, C4, C5 and C9) and their membrane regulators (decay-accelerating factor, membrane cofactor protein (DAF9, and CD59) [70]. Herein, C3a and C5b9 were assayed in vitreous, and their expression was correlated with ERM severity. TLR2 was found to induce complement factors C3 [35]. TLR2 deficiency preserves tight junction expression and promotes RPE resistance to fragmentation [35]. The neutralization of TLR2 reduces the opsonizing fragments of C3 in the outer retina and protects the photoreceptor neurons from oxidative stress-induced degeneration [35]. The oxidative stress-induced formation of the terminal complement membrane attack complex and the infiltration of Iba1-positive cells are strikingly inhibited in TLR2-deficient retinas [35]. In previous studies, TLR2 was reported as a mediator of retinal degeneration in response to oxidative stress and was hypothesized to act as a “bridge” between oxidative damage and complement-mediated retinal pathology. 

The limitations of this study are represented by the following: i. the absence of data on the quantification of soluble TLR2 and TLR4 forms and the detection of C5aR in ERMs, ii. a limited number of specimens for chip-array analysis and correlation studies and finally, iii. the presence of some risk factors that might influence innate immune response, such as gender, ageing and some major metabolic comorbidities [71,72,73,74,75]. The small study population is in line with the observation that 2% of middle-aged individuals (over age 50) and 20% of elderly individuals (over age 75) show evidence of ERMs, although they do not need treatment [1]. The gender bias might also represent a limitation of this study, although age/sex matched cases and controls were compared. Although recognized as an immune privilege site, the eye might respond to changes in the sexual hormones and ageing. The hormonal levels can influence/modulate the immune response locally, and particularly females can display a higher immune response than age-matched males [73]. On the other hand, ageing is also known to influence circulating and tissue-specific hormonal levels [74]. In fact, immune parameters decline at a slower rate in aged women, although genome activity remains higher for acquired immunity than for innate immunity. On the other hand, men exhibit higher gene activity in innate immunity [74]. Certainly, our study populations included biosamples collected from patients having risk factors related to vascular impairments and/or metabolic, autoimmune and allergic disorders, while a small population of patients presented with an apparent good health condition. Previous studies suggest that the ERMs are relatively common among the aged population, and the potential risk factors include few but major metabolic comorbidities, such as hypertension, type 2 diabetes and hypercholesterolemia/dyslipidemia, in addition to age and sex [75]. The association of TLR2 and TLR4 with allergies is in line with previous studies, so we cannot exclude such influences on the modulation of TLRs in ERM severity [74]. Our preliminary analysis on the influence of the major comorbidities (clustered as with or without hypertension) on TLR expression suggests the need for a more comprehensive analysis related to ERM formation or recurrence, considering these metabolic influences. While it is well established that TLRs participate in the inflammatory process aimed at PAMPs’ and/or DAMPs’ removal, TLRs’ involvement at the beginning and progression of chronic pathologies appears to be just a possibility [75]. 

## 4. Materials and Methods

This study was approved by the intramural ethical committee (IFO-Bietti, Rome, Italy) and performed in accordance with the ethical standards stated in the Declaration of Helsinki. The patients approved the experimental approach and signed the consent forms for specimen collection, handling and analysis. 

### 4.1. Study Population, SD-OCT Classification and Subgrouping

ERM (20 peeled-off membranes) and vitreal (12 collected samples) specimens were sampled from 20 patients (16F/4M; 71.45 ± 7.36 years old) and underwent vitrectomy with peeling of epiretinal membranes. Before surgery, a full ophthalmic examination was carried out, including anamnesis, funduscopic evaluation, and Spectral Domain–Optical Coherence Tomography (Spectralis SD-OCT ver.1.5.12.0; Heidelberg Engineering, Heidelberg, Germany) [25,26]. The ERM classification was as follows: stage 1, mild and thin ERMs with presence of foveal depression; stage 2, ERMs associated with widening of nuclear layer and loss of foveal depression; stage 3, ERMs associated with continuous ectopic inner foveal layers crossing the entire foveal area; and stage 4, thick ERMs associated with continuous ectopic inner foveal layers and severe disruption of retinal layers [25]. Inclusion criteria were stage 2, stage 3 and stage 4 (subgrouping). Exclusion criteria comprised stage 1 ERMs (patients with anatomical and morphological retinal status do not need surgery), macular holes, proliferative diabetic retinopathy, age-related macular degeneration, retinal vascular occlusion, aphakia, high myopia (≥28 diopters), uncontrolled glaucoma. Additional exclusion criteria were prior vitrectomy, intravitreal injections and retinal laser photocoagulation, as well as opacity of optical media. The characteristics of the study population and specimen collection are summarized in Table 1. The chart also shows paired vitreal samples whenever obtained. 

### 4.2. Sampling Mode and Pre-Analytics

The procedure for sampling included the vitreous collection at the beginning of standard 23G pars plana vitrectomy, just before opening the infusion port, and subsequent ERM peel-off. Untouched vitreous was delivered to the laboratory and processed as described below. 

ERMs were placed as whole-mounted tissues on pretreated glass slides (BDH, Milan, Italy), postfixed (ThinPrep^®^ PreservCyt solution, Hologic, Inc., Marlborough, MA, USA) and probed for immunofluorescent analysis. For Real-Time PCR and IP/WB analyses, ERMs were extracted in 300 μL of lysis solution (MirVana Paris™ Invitrogen; Thermo Fisher Scientific, Waltham, MA, USA) and native protein, as well as total RNA, were obtained from each tissue/section.

### 4.3. Double Immunofluorescent Analysis and Digital Acquisitions

A total of 20 ERMs (N = 8 for stage 2, 6F/2M; N = 6 for stage 3, 5F/1M; N = 6 for stage 4, 5F/1M) were used for immunofluorescent analysis. Briefly, whole-mounted ERMs were briefly rehydrated in PBS (10 mM Phosphate Buffer (PB) and 137 mM NaCl; pH 7.5). A blocking/permeabilizing step (0.1% BSA and 0.3% Triton X100 in PBS) was carried out before antibody incubation: anti-human TLR2 antibody (mouse; 1:200; Santa Cruz, CA, USA), anti-human TLR4 antibody (rabbit; 1:200; Santa Cruz), anti-human α-SMA antibody (mouse; 1:500; Sigma-Aldrich, Saint Louis, MO, USA), anti- Iba1 antibody (mouse; 1:500; Santa Cruz) and Anti- GFAP antibody (mouse; 1:500; Cell Signaling, Denver, MA, USA). Specific bindings were detected using secondary AlexaFluor-555 or AlexaFluor-488-coupled anti-rabbit (for TLR4) or anti-mouse (for TLR2, α-SMA, Iba1, GFAP) specie-specific F(ab)2 antibodies diluted (1:500) in 0.05% Tween20-PBS and incubated for 45 min on benchtop (Immunological Sciences, Rome, Italy). After nuclear counterstaining (blue/DAPI; Invitrogen Molecular Probes, MA, USA), the slides were mounted with an anti-fading supplemented Vectashield (Vector Laboratories, Inc., Burlingame, CA, USA) and examined under an epifluorescent direct microscope (Ni-Eclipse) equipped with UV lamp, digital camera (Axiocam) and NIS-Elements software F 4.00.00 for digital assets (8-bit TIFF format; Nikon, Konan, Minato-ku, Tokyo). Acquisitions were carried out with ×200 and ×400 objectives, both in single and merged image sets.

### 4.4. Molecular Analysis: Total RNA, cDNA and Relative Real-Time PCR

Twenty ERMs previously immunostained were used for molecular analyses. Briefly, total RNAs were extracted according to the MirVana Paris procedure with minor modifications, rehydrated in 11 μL RNase-free water (DEPC-treated/autoclaved MilliQ water, Sigma-Aldrich) and spectrophotometrically checked for RNA quantity/purity (>1.8; A280 program, Nanodrop; Celbio, Euroclone S.p.A, Milano, Italy). For cDNA synthesis, 100 ng of total RNA was retro-transcribed using the GoScript RT mix and random hexamers (Promega, Madison, WI, USA) in a LifePro Thermal Cycler (Euroclone, Rome, Italy). For amplification, 3 μL (target gene) and 1 μL (referring gene) cDNA were amplified with the SYBR-green hot-start PCR master mix (Hydra Taq; Biolab; Biocell, Rome, Italy) using the Eco™ Real-Time PCR platform (Illumina, San Diego, CA, USA). 

Amplifications were carried out for both target/referring genes per sample and in parallel with negative/positive controls. PCR products (100–200 bps) were separated in 2.5% agarose gel (mini horizontal apparatus; Bio-Rad, Hercules, CA, USA), and bands were observed/acquired inside the UVP station (TIFF-format images). Cycle threshold values (Cq) from normalized samples with one melting curve were used for REST analysis [76]. Changes in target gene expression observed at stage 3 and stage 4 were provided as log2 expression ratio with respect to stage 2 (herein considered the reference subgroup), considering H3 as the reference gene. Primer pairs were synthesized by Eurofin MWG Genomics (www.eurofinsgenomics.eu, accessed on 29 march 2023), with at least one intron spanning primer (Primer3 Input (version 0.4.0)), and the sequences are reported in Supplementary Table S1.

### 4.5. Biochemical Analysis: IP/WB, ELISA and Chip-Based Protein Array

A total of 12 vitreous (N = 5 for stage 2, 3F/2M; N = 4 for stage 3, 3F/1M; N = 3 for stage 4, 2F/1M) were used for biochemical analyses. Briefly, the samples (250–500 μL) were quickly stabilized with protease inhibitors (1 µL/sample; Pierce, Thermo Fisher Scientific) and split into two aliquots that were allocated to IP/WB—ELISA and chip array analysis, as described below. Aliquots for IP/WB—ELISA were sonicated (VibraCell; Sonics, Newtown, CT, USA) and centrifuged to remove debris (13,000 rpm/7 min; Sigma 1–14 microfuge), while sister aliquots for chip array were centrifuged (2000 rpm for 7 min; Sigma 1–14 microfuge) to separate floating cells. The clarified supernatants were further sonicated (VibraCell, Newtown, CT, USA) and centrifuged to remove debris (13,000 rpm/7 min). A spectrophotometer analysis (3 μL) was carried out (N1000, Nanodrop) before producing aliquots.

*IP/WB.* The captured antibodies of interest (Eotaxin, Santa Cruz; GFAP, Immunological Sciences; TLR2, Santa Cruz and TLR4, Santa Cruz) were first immobilized onto the Pure Proteome Protein G Magnetic beads (15 µL, Millipore, Burlington, MA, USA) to generate the antibody–beads complex. The beads-bound antibodies were then added to the normalized samples (100 µg total protein) and after 2 h of incubation, the captured proteins were washed and eluted in denaturing loading buffer. All steps were performed under orbital shaking (Certomat II, Sartorius, D-72393 Burladingen, Deutschland). LB samples were preheated at 90 °C for 10 min and loaded on 4–12% precasted SDS-PAGE gels (Bio-Rad Laboratories Inc., Hercules, CA, USA), and electrophoresis was performed in a MiniProtean3 apparatus (Bio-Rad) under reducing conditions (120 V/frontline).

Electrophoresed bands were transferred to 0.22 µm membranes (Hybond, GE Healthcare, Buckinghamshire, UK) at 12 V/40 min in a semidry Trans-Blotting apparatus (Bio-Rad). The membranes were stained with the high-sensible Sypro Ruby protein blot stainer (Invitrogen, Waltham, MA, USA), according to the standard procedure, to visualize and acquire the separations. Immunoblotting, followed by chemiluminescent detection, was additionally performed to better visualize the proteins of interest. In both cases, optical density (OD) was performed using the freely available ImageJ software (software 1.54j, National Institutes of Health, Bethesda, MD, USA). Data were saved as 8-bit TIFF files and exported for figure assembly using the Adobe Photoshop 2024 program (Release 25.5.1, Adobe Systems, Inc., Mountain View, CA, USA). The loading of normalized samples was verified by checking the *β*-actin content (predicted molecular weight: 40–42 kDa; sc-47778, Santa Cruz). 

*ELISA*. Aliquots (50 μL/sample) were 1:2 diluted in a sample dilution buffer provided by commercially available C3a and C5b-9 ELISA kits (Cloud-Clone Corp., Houston, TX, USA), including precoated 96-well plates and ready-to-use solutions. All incubation and washing procedures were performed according to the manufacturers’ suggestions, with minor modifications. Absorbance (optical density—OD) values were recorded after plate reading (λ450–λ570 nm) and concentrations were produced according to standard curves (assay range: 5000 pg/mL–78 pg/mL for C3a and 80 ng/mL–1.25 ng/mL for C5b-9).

*Chip-Based Protein Array.* Sister aliquots were analyzed in customized G-series glass slides (14 identical subarrays/slide with 57 targets; RayBiotech, Norcross, CA, USA). Array chips were incubated with prediluted vitreous samples and labelled with a biotin-conjugated cocktail of antibodies, followed by a Cy3-conjugated streptavidin complex, according to the manufacturer’s instructions. As a final point, the glass slides were washed once with MilliQ water, spin-dried (700 g/1 min) and scanned using a GenePix 4400 Microarray scanner (Molecular Devices LLC, Sunnyvale, Silicon Valley, CA, USA). The fluorescence intensity data (FI) were generated by GenePix Pro 6.0 software (Axon Instruments, Foster City, CA, USA) and provided as background-subtracted FI data (F532-B532, N factor) for each spot/volume. Values were expressed as ratio (pathological vs. reference signal). Inter-assay normalization was guaranteed by the presence of multiple internal controls for each subarray. To obtain appropriate Cy5 (background signal) and Cy3 (specific signal) images, the slides were scanned over previously validated acquisition parameters and the images/arrays (blocks) were uniformly adjusted for size, brightness and contrast at the time of acquisition. 

### 4.6. Statistics 

Data were first analyzed using the Kolmogorov–Smirnov and the Shapiro–Wilk tests to satisfy the assumption of values coming from a normally distributed population (Prism10.0; GraphPad Software Inc., San Diego, CA, USA). The one-way ANOVA analysis was used to compare protein expression between the subgroups. The REST–ANOVA coupled analysis was carried out for identifying significant changes in Real-Time PCR experiments. Correlations were assessed between subgroups using the Pearson rho correlation test. Some statistical analyses were confirmed with RStudio (Version: 2024.04.2+764, open-source platform). The two-sided unpaired *t*-test comparisons or the Wilcoxon–Mann–Whitney test followed by the Benjamini–Hochberg procedure were used for multiple-test corrections. Significance levels were * *p* < 0.05, ** *p* < 0.01, *** *p* < 0.001 and **** *p* < 0.0001.

Continuous variables were reported as mean values and standard deviations, and categorical variables were represented as numbers and percentages. The statistical tests used in this study were the independent-sample *t*-test, Chi-squared test, Mann–Whitney U-test and Spearman correlation, as appropriate.

## 5. Conclusions

Corroborating studies suggest that the presence of innate immune cells and chronic low-grade inflammation might influence the development, function and evolution of ERMs and even their reappearance [77]. TLRs have the primary role to detect ‘non-self’ pathogen molecules, sensor endogenous tissue-damaged host-derived molecules (DAMPs) and guarantee tissue homeostasis. TLRs work in combination with complement fragments, ameliorating some inflammatory states linked to DAMPs’ recognition, although exacerbations of the local inflammation and senescence have also been reported [31,78,79]. Our findings on the presence of TLR2 and TLR4 in these ERMs and their specific expression by contractile α-SMA-expressing cells, Iba1-immunoreactive microglial cells and GFAP-positive Müller cells would suggest the participation of innate response in the evolution and contraction abilities of ERMs [41,42,43,44,45,46,47]. Since the possibility of impaired TLR/DAMP signaling cannot be excluded, the TLR/complement system and its modulation might be, in the near future, an additional intravitreal target, especially to counteract recidivism. The ERM system might be an interesting “ex vivo” model to assess the inflammatory status and the cross-talk between the innate and adaptive response. Although vitrectomy and ERM peel-off continue to be the first-choice therapy for vitreoretinal diseases, much remains to be investigated regarding the development of alternative “drug” therapies for the prevention and early treatment of vitreous–retinal interface syndromes that are not associated with traumatic events. Further studies are underway to better elucidate these aspects, which could be useful in the near future for individualized therapies (personalized medicine).

## Figures and Tables

**Table 1:** Study population and biosamples.

ID	Gender (M/F)	Age (Years)	Vitreous (Y/N)	ERM (Y/N)	Staging (Grades)	Comorbidities (List)
1	F	71	Y	Y	4	Hypertension, Cardiopathy
2	M	54	Y	Y	2	Hypertension, Cardiopathy, Type 2 Diabetes
3	F	63	N	Y	2	Hypercholesterolemia, Keratoconus
4	M	78	Y	Y	4	Hypertension, Hypercholesterolemia
5	F	69	N	Y	4	Hypercholesterolemia
6	F	66	N	Y	3	None
7	F	72	N	Y	2	None
8	F	70	Y	Y	3	Hypertension, Hypothyroidism
9	F	69	N	Y	3	Asthma, Thyroidectomy
10	F	65	Y	Y	4	Hypercholesterolemia, Type 2 Diabetes
11	F	83	Y	Y	2	Hypercholesterolemia
12	F	82	N	Y	4	Hypertension, Hypercholesterolemia
13	F	80	Y	Y	2	Hypertension, Cardiopathy, Type 2 Diabetes
14	F	69	Y	Y	2	Hypertension, Type 2 Diabetes, Hyperuricemia
15	F	81	Y	Y	3	Hypertension, Asthmatic Bronchitis
16	M	66	Y	Y	3	Type 2 Diabetes
17	F	71	Y	Y	3	Hypertension, Hypercholesterolemia
18	F	80	N	Y	2	Muscular Dystrophy
19	M	71	Y	Y	2	Hypertension, Asthma
20	M	69	N	Y	4	None

The chart summarizes demographic aspects of study population, sampling aspects (paired and unpaired aspects of ERM/vitreous samples) and comorbidities. Complete ophthalmic examination and ERM grading were assessed by two ophthalmologists (A.C. or G.R.). Visual acuity progressively declined from stage 1 to stage 4 [25]. Legend: ID, patients’ progressive code; gender (M/F, male/female); age (years); vitreous (Y/N, yes/no); ERM (Y/N, yes/no); staging (grades); comorbidities, as depicted from initial anamnesis (list).

## Data Availability

All data generated or analyzed during this study are included in this published article.

## Supplementary Materials

The supporting information can be downloaded at: https://www.mdpi.com/article/10.3390/ijms25147732/s1.
