# Supplementary material for: TLR2 and TLR4 Are Expressed in Epiretinal Membranes: Possible Links with Vitreous Levels of Complement Fragments and DAMP-Related Proteins
Source: Int J Mol Sci. 2024 Jul 15;25(14):7732. doi: 10.3390/ijms25147732 (PMC11276880; doi:10.3390/ijms25147732)
Supplement: Supplementary file 1 [file ijms-25-07732-s001.zip › ijms-3050682-supplementary.pdf]

Table S1 List of primers

Name	*Accession number*	*Sequences (5′-3′)*	*C° (Tm)*	*bps*
A) Targets				
**TLR2**	BC033756.1	F: TGA TGC TGC CAT TCT CAT TC<br>R: CGC AGC TCT CAG ATT TAC CC	59.7<br>59.9	157
**TLR4**	U88880.1	F: TGT CCT GCA GAA GGT GGA G<br>R: CAG GGC TTT TCT GAG TCG TC	59.6<br>59.6	139
**MyD88**	AF406652.1	F: AGC GAT GCT TCA CAG CCT AC<br>R: CTC CAG CGA CTA CTG CCA CT	59.4<br>61.4	116
**p65-NFkB**	L19067.1	F: CAG AAG CAG GCT GGA GGT AA<br>R: GTT AGG CAC AGG GAC AAT GC	59.4<br>59.4	140
**MMP1**	BC013875.2	F: TCC CAG AGA GCA GCT TCAGT<br>R: CCT ATC CAG GGT GAC ACC AG	59.4<br>61.4	127
**MMP2**	BC002576.2	F: TCA GAG CCA CCC CTA AAG AG<br>R: CTG TGA GCC ACA GAA GGT TG	59.4<br>59.4	115
**MMP3**	BC069716.1	F: ACC TGT CCC TCC AGA ACC T<br>R: CGC CAA AAG TGC CTG TCT	59.4<br>56.0	115
**MMP7**	BC003635.2	F: GAGCTCATGGGGACTCCTAC<br>R: ACT GCT ACC ATC CGT CCA G	61.4<br>58.8	100
**MMP9**	BC006093.1	F: CCA GGA GTT CGA GGC TGT AG<br>R: TCC CAA ACC ACA GGA CTT TC	61.4<br>57.3	113
**TIMP1**	BC000866.1	F: GGC TGT GAG GAA TGC ACA GT<br>R: CCC TTT TCA GAG CCT TGG AG	59.4<br>59.4	107
**TIMP2**	NM_003255.5	F: GTG GGT CCA AGG TCC TCA T<br>R: CTA AGT GCT GCC TGC TTG G	58.8<br>58.8	100
**HLA-DR**	NM_019111.5	F: CCTGTCACCACAGGAGTGTC<br>R: GAGAAGAGGCTCATCCAAGC	61.4<br>59.4	147
B) House-keeping				
**Histon H3**	NM_005324.3	F: GTCTGCAGGCTGGCATAGAAG<br>R: TCGCCTTCTGGGTTGAGTG	61.8<br>58.8	110

Specific amplifications were tested by verifying the single curve specific for each amplicon. Hot-start SYBRgreen mix was activated by a pre-hold (5min at 50°C) and pre-incubation for 5min at 95°C. Each of the 39 amplification cycles consisted of a 30sec/94°C (denaturation), followed by a specific annealing step (appropriate temperature, ~Tm – 3°C, verified for specificity by grading) and 30sec/72°C (extension). Melting curve was registered from 56.0°C to 94.1°C; 0.3°C; hold for 00:00:01 between reads.
